# *Bundibugyo ebolavirus* Survival Is Associated with Early Activation of Adaptive Immunity and Reduced Myeloid-Derived Suppressor Cell Signaling

**DOI:** 10.1128/mBio.01517-21

**Published:** 2021-08-10

**Authors:** Courtney Woolsey, Viktoriya Borisevich, Krystle N. Agans, Karla A. Fenton, Robert W. Cross, Thomas W. Geisbert

**Affiliations:** a Galveston National Laboratory, University of Texas Medical Branch, Galveston, Texas, USA; b Department of Microbiology and Immunology, University of Texas Medical Branch, Galveston, Texas, USA; Icahn School of Medicine at Mount Sinai

**Keywords:** coagulation, Ebola virus, filovirus, immunology, myeloid-derived suppressor cell, nonhuman primate, pathogenesis

## Abstract

Ebolaviruses Bundibugyo virus (BDBV) and Ebola virus (EBOV) cause fatal hemorrhagic disease in humans and nonhuman primates. While the host response to EBOV is well characterized, less is known about BDBV infection. Moreover, immune signatures that mediate natural protection against all ebolaviruses remain poorly defined. To explore these knowledge gaps, we transcriptionally profiled BDBV-infected rhesus macaques, a disease model that results in incomplete lethality. This approach enabled us to identify prognostic indicators. As expected, survival (∼60%) correlated with reduced clinical pathology and circulating infectious virus, although peak viral RNA loads were not significantly different between surviving and nonsurviving macaques. Survivors had higher anti-BDBV antibody titers and transcriptionally derived cytotoxic T cell-, memory B cell-, and plasma cell-type quantities, demonstrating activation of adaptive immunity. Conversely, a poor prognosis was associated with lack of an appropriate adaptive response, sustained innate immune signaling, and higher expression of myeloid-derived suppressor cell (MDSC)-related transcripts (*S100A8*, *S100A9*, *CEBPB*, *PTGS2*, *CXCR1*, and *LILRA3*). MDSCs are potent immunosuppressors of cellular and humoral immunity, and therefore, they represent a potential therapeutic target. Circulating plasminogen activator inhibitor 1 (PAI-1) and tissue plasminogen activator (tPA) levels were also elevated in nonsurvivors and in survivors exhibiting severe illness, emphasizing the importance of maintaining coagulation homeostasis to control disease progression.

## INTRODUCTION

The *Ebolavirus* genus comprises six species: *Zaire ebolavirus*, *Bundibugyo ebolavirus*, *Sudan ebolavirus*, *Tai Forest ebolavirus*, *Reston ebolavirus*, and the recently discovered *Bombali ebolaviru*s (1). The ongoing outbreaks of Ebola virus (EBOV; species *Zaire ebolavirus*) in the Democratic Republic of Congo and Guinea demonstrate that ebolaviruses continue to pose a significant threat to human health (2, 3). Clinical manifestations of ebolavirus-infected humans and nonhuman primates (NHPs) are similar, including high viremia, hypercytokinemia, and consumptive coagulopathy, which may progress to septic shock and multiorgan failure (4, 5). The virus initially replicates in monocytes and dendritic cells and then spreads to hepatocytes, endothelial cells, and epithelial cells. Bundibugyo virus (BDBV; species *Bundibugyo ebolavirus*) is considered a less pathogenic ebolavirus due to its reduced lethality during human outbreaks (25 to 51%), as well as in experimentally infected cynomolgus and rhesus macaques (4, 6–8). In contrast, infection of NHPs with EBOV results in nearly uniform lethality (4). While in-depth transcriptome analyses have been performed on samples from EBOV-infected human patients and NHPs, no such studies exist for BDBV (9–16). As macaques are incompletely protected against BDBV disease, we reasoned that this experimental model could aid in the identification of specific immune cell populations and transcriptional correlates that support natural defense.

In this study, longitudinal whole blood samples were collected from BDBV-infected rhesus macaques. Clinical pathology, viral loads, and plasma levels of cytokines, chemokines, and thrombosis markers were assessed in surviving and nonsurviving animals (referred to as survivor and fatal subjects, respectively). Transcriptional changes in each data set were compared at early, middle, and late time points after infection to define the immune response at each stage of disease. Humoral responses were measured with BDBV glycoprotein (GP)-specific IgM and IgG enzyme-linked immunosorbent assays (ELISAs) and plaque reduction neutralization tests (PRNTs). These analyses characterize the systemic host response to BDBV exposure. Here, we demonstrate that early cellular and humoral immune responses contribute to survival, whereas prolonged innate immune signaling, coagulation anomalies, and myeloid-derived suppressor cell-associated signaling are associated with severe or fatal disease.

## RESULTS

### Experimental infection of rhesus macaques.

**FIG 1:**
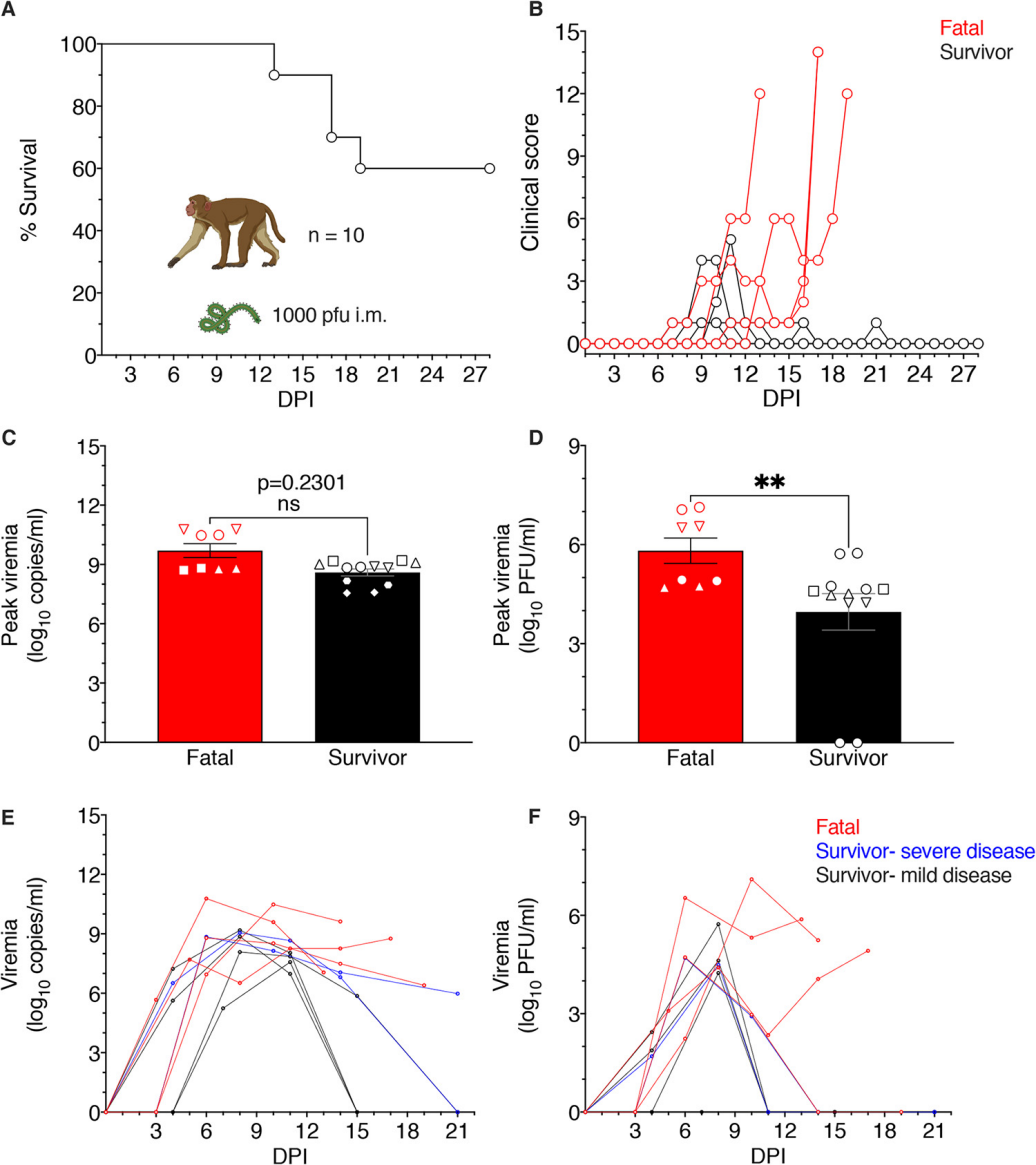
Survival of BDBV-infected macaques and comparison of peak viral loads and lesion severity scores in fatal and surviving subjects. (A) Survival curve of rhesus macaques (*n* = 10) infected intramuscularly with 1,000 PFU of BDBV-Uganda up to the ≥28-day endpoint. (B) Clinical scores of individual fatal versus surviving BDBV-infected macaques; criteria include behavior, posture and activity level, appetite, respiration, and the presence of hemorrhagic manifestations. (C) Peak viral loads were measured by RT-qPCR in whole blood and reported as log_10_ copies/ml irrespective of the day on which the highest viremia was detected. The limit of detection for this assay was 1,000 copies/ml. (D) Peak viral loads were measured in plasma samples by standard plaque assay and reported as log_10_ PFU/ml irrespective of the day on which the highest viremia was detected. The limit of detection for this assay was 25 PFU/ml. (C and D) Statistical significance was determined using the Mann-Whitney nonparametric two-tailed *t* test. ns, no statistically significant difference; ** *P* < 0.001; ***, *P* < 0.0001; ****, *P* < 0.00001. (E and F) Viral loads determined by PCR (E) or plaque assay (F) at each dpi sampled are displayed and are reported as log_10_ copies/ml or log_10_ PFU/ml, respectively. Red denotes fatal group, black denotes survivor group. Each replicate is shown with symbols denoting data for individual subjects (*n* = 10 biologically independent animals/samples per tissue type in a single experiment); each bar and error bar represents the group mean value ± the standard error of the mean (SEM).

Ten adult rhesus macaques were intramuscularly (i.m.) inoculated with a 1,000-PFU target dose of BDBV. The survival rate of macaques up to the 28-day-postinfection (dpi) study endpoint was ∼60% (6 of 10 animals) (Fig. 1A). Of the four fatal cases, one animal succumbed at 13 dpi, two animals succumbed at 17 dpi, and one animal succumbed at 19 dpi. Two survivors (survivor 1 and survivor 2) had clinical scores of ≥4 at 9 to 11 dpi, but their condition rapidly improved by 12 dpi (Fig. 1B). All BDBV-infected macaques developed various degrees of illness, including fever, anorexia, macular rash, and/or depression (Table 1). Disease manifestations in the fatal cohort were generally more severe. All infected macaques developed fevers except fatal 4 and survivor 5, and all animals except survivor 4 experienced anorexia. Three of four fatal subjects developed a mild petechial rash, one exhibited facial edema, three had diarrhea, and one animal showed signs of neurological disease (fatal 4). In the survivor cohort, three of five survivors suffered mild petechial rashes and one subject presented with emesis and mild dehydration (survivor 2). Hematological or serum biochemistry changes were evident in all BDBV-infected macaques. Fatality correlated with the onset of thrombocytopenia, lymphopenia, and granulocytosis, although these cell population changes were also transiently observed in some survivors. All animals except survivor 5 had elevated levels of liver enzymes, including alanine aminotransferase (ALT), aspartate aminotransferase (AST), alkaline phosphatase (ALP), and gamma-glutamyltransferase (GGT). Increased blood urea nitrogen (BUN) and/or creatinine (CRE) concentrations were prominent in the serum of fatal cases and two survivors, pointing to potential kidney damage. C-reactive protein (CRP) levels were also increased in all fatal cases and four survivors, indicating systemic inflammation.

**TABLE 1:** Clinical findings of *Bundibugyo ebolavirus*-infected rhesus macaques

Animal ID (sex), wt^*a*^	Titer (log_10_ copies/ml) (dpi) determined by^*b*^:		Clinical signs (dpi)^*b*^^,^^*c*^	Final outcome
	RT-qPCR	Viremia assay		
Fatal 1 (M), 5.82 kg	6.95 (6), 10.48 (10), 9.62 (14)	2.24 (6), 7.10 (10), 5.24 (14)	Fever (6), anorexia (10), mild depression (10–16), moderate depression (13), severe depression (17), facial edema (15–16), thrombocytopenia (10, 14), lymphopenia (6, 14), granulocytosis (3, 6, 10, 14), BUN ++ (10, 14), ALT ++ (14) +++ (10), AST +++ (10, 14), ALP + (10) ++ (14), GGT ++ (10, 14), CRP increase (6, 10)	Succumbed at 17 dpi
Fatal 2 (M), 6.98 kg	8.79 (6), 8.52 (10), 7.49 (14), 6.40 (19)	4.72 (6), 2.97 (10)	Fever (6), anorexia (10), mild petechial rash (9–12), mild depression (7), moderate depression (8–19), mildly swollen axillary and submandibular lymph nodes (6), diarrhea (10, 11, 14), thrombocytopenia (10), lymphopenia (6), monocytosis (10), granulocytosis (14), BUN + (10) ++ (14), ALT ++ (10), AST +++ (10, 14), CRP increase (6, 10, 14)	Succumbed at 19 dpi
Fatal 3 (M), 6.16 kg	5.67 (3), 10.78 (6), 9.59 (10), 7.05 (13)	6.53 (6), 5.32 (10), 5.88 (13)	Fever (6), anorexia (7), mild petechial rash (10–13), moderate depression (10–13), diarrhea (11–13), leukocytosis (10, 13), thrombocytopenia (10, 13), lymphopenia (6), monocytosis (10, 13), granulocytosis (6, 10, 13), BUN +++ (10, 13), CRE ++ (13), ALT ++ (6) +++ (10, 13), AST +++ (6, 10, 13), GGT + (6) ++ (13) +++ (10), CRP increase (6, 13)	Succumbed at 13 dpi
Fatal 4 (F), 2.64 kg	7.70 (5), 6.52 (8), 8.26 (11), 8.26 (14), 8.76 (17)	3.09 (5), 4.41 (8), 2.35 (11), 4.06 (14), 4.92 (17)	Anorexia (4, 11, 12, 15–17), mild petechial rash (14–17), mild depression (11–15), moderate depression (16), severe depression (17), tremor (14–17), diarrhea (0–17), leukopenia (5, 8, 14, 17), thrombocytopenia (11, 14), lymphopenia (8), BUN + (5) ++ (14, 17), ALT ++ (11, 14) +++ (17), AST +++ (11, 14, 17), GGT + (17), CRP increase (5)	Succumbed at 17 dpi
Survivor 1 (M), 5.22 kg	8.85 (6), 8.14 (10), 7.05 (14), 5.98 (21)	4.70 (6), 2.92 (10)	Fever (6, 14), anorexia (10), mild petechial rash (9–11), mild depression (7, 11, 17–19), moderate depression (8–10), mildly swollen submandibular lymph nodes (6), tremor (17–21), leukocytosis (21), thrombocytopenia (10), monocytosis (21), granulocytosis (6, 10, 14, 21), BUN ++ (14) +++ (10), CRE +++ (10), ALT +++ (10), AST +++ (10), ALP + (10, 14), GGT ++ (10), CRP increase (6, 10, 19)	Survived
Survivor 2 (F), 4.20 kg	6.51 (4), 9.05 (8), 8.66 (11), 6.81 (14)	1.70 (4), 4.49 (8)	Fever (8), anorexia (10, 11), mild depression (10–16), emesis (10), mild dehydration (14–16), diarrhea (12–14), leukocytosis (4, 21), thrombocytopenia (11, 14), monocytosis (4), granulocytosis (4, 21), BUN + (11, 14), ALT ++ (8) +++ (11, 14), AST +++ (8, 11, 14), GGT + (11)	Survived
Survivor 3 (F), 4.74 kg	5.63 (4), 8.86 (8), 6.98 (11)	4.24 (8)	Fever (8), anorexia (9, 10), mild petechial rash (8–13), leukopenia (8, 11), thrombocytopenia (4, 8, 11, 15), lymphopenia (8), ALT + (8), AST + (11) +++ (8), CRP increase (8)	Survived
Survivor 4 (M), 4.28 kg	7.23 (4), 9.18 (8), 8.04 (11)	1.88 (4), 4.62 (8)	Fever (8), mild petechial rash (11, 12), leukocytosis (15), thrombocytopenia (8, 11), monocytosis (4), granulocytosis (15), ALT + (15) ++ (11) +++ (8), AST +++ (8, 11), CRP increase (8)	Survived
Survivor 5 (F), 4.54 kg	5.24 (7), 7.57 (11)		Anorexia (7), leukopenia (7, 21)	Survived
Survivor 6 (F), 6.16 kg	8.08 (8), 7.86 (11), 5.86 (15)	2.44 (4), 5.73 (8)	Fever (8), anorexia (9, 10), thrombocytopenia (11), ALT + (8) ++ (11), AST ++ (8, 11), CRP increase (8)	Survived

a M, male; F, female.

b Days after BDBV challenge up to 21 dpi are in parentheses.

c Fever is defined as a temperature greater than 2.5°F above baseline, at least 1.5°F above baseline and ≥103.5°F, or 1.1°F above baseline and ≥104°F. Leukopenia, thrombocytopenia, and lymphopenia are defined by a >40% drop in the number of leukocytes, platelets, or lymphocytes, respectively. Leukocytosis, monocytosis, and granulocytosis are defined as a ≥2-fold increase in leukocytes, monocytes, or granulocytes, respectively. Plus signs indicate increases in liver enzymes (ALT, AST, ALP, and GGT) or renal function test values (BUN and CRE) as follows: +, 2- to 3-fold increase; ++, >3- up to 5-fold increase; +++, >5-fold increase. BUN, blood urea nitrogen; CRE, creatinine; ALT, alanine aminotransferase; AST, aspartate aminotransferase; ALP, alkaline phosphatase; GGT, gamma-glutamyltransferase; CRP, C-reactive protein; dpi, days postinfection.

### Circulating viral loads in the plasma of infected macaques.

We assessed the levels of viremia in each cohort by performing reverse transcriptase quantitative PCR (RT-qPCR) amplification of viral genomic RNA and conventional plaque assays on whole blood samples. All infected animals were PCR positive (Fig. 1C and Table 1). Average peak PCR titers were 9.700 ± 0.998 log_10_ copies/ml (mean ± standard error of the mean [SEM]) for fatal cases, whereas the mean titers for survivors were 8.596 ± 0.610 log_10_ copies/ml. Interestingly, the peak viral RNA titers were not significantly different between surviving and nonsurviving subjects (Mann-Whitney nonparametric two-tailed *t* test, *P* = 0.2301). However, the average peak infectious viral loads were significantly different between the two groups (Mann-Whitney nonparametric two-tailed *t* test, *P* = 0.0017), with titers of 5.815 ± 1.090 and 3.962 ± 1.914 log_10_ PFU/ml for nonsurviving and surviving subjects, respectively (Fig. 1D and Table 1). Infectious virus was never detected in survivor 5 at the time points tested, but this animal had detectable viral RNA on days 7 (5.24 log_10_ copies/ml) and 11 (7.57 log_10_ copies/ml) (Table 1). The high PCR titers and absence of detectable infectious virus in this animal may indicate rapid clearance of viable virus with noninfectious virus components persisting in the blood (17). Viremia was reduced or absent in 3 of 4 fatal subjects at end-stage disease, suggesting immune-mediated rather than virus-induced pathology. Finally, no ostensible trend between nonsurviving and surviving (mild or severe) subjects was apparent for viral RNA (Fig. 1E) or infectious virus (Fig. 1F) loads over the course of the study.

### Histopathology and immunohistochemistry.

**FIG 2:**
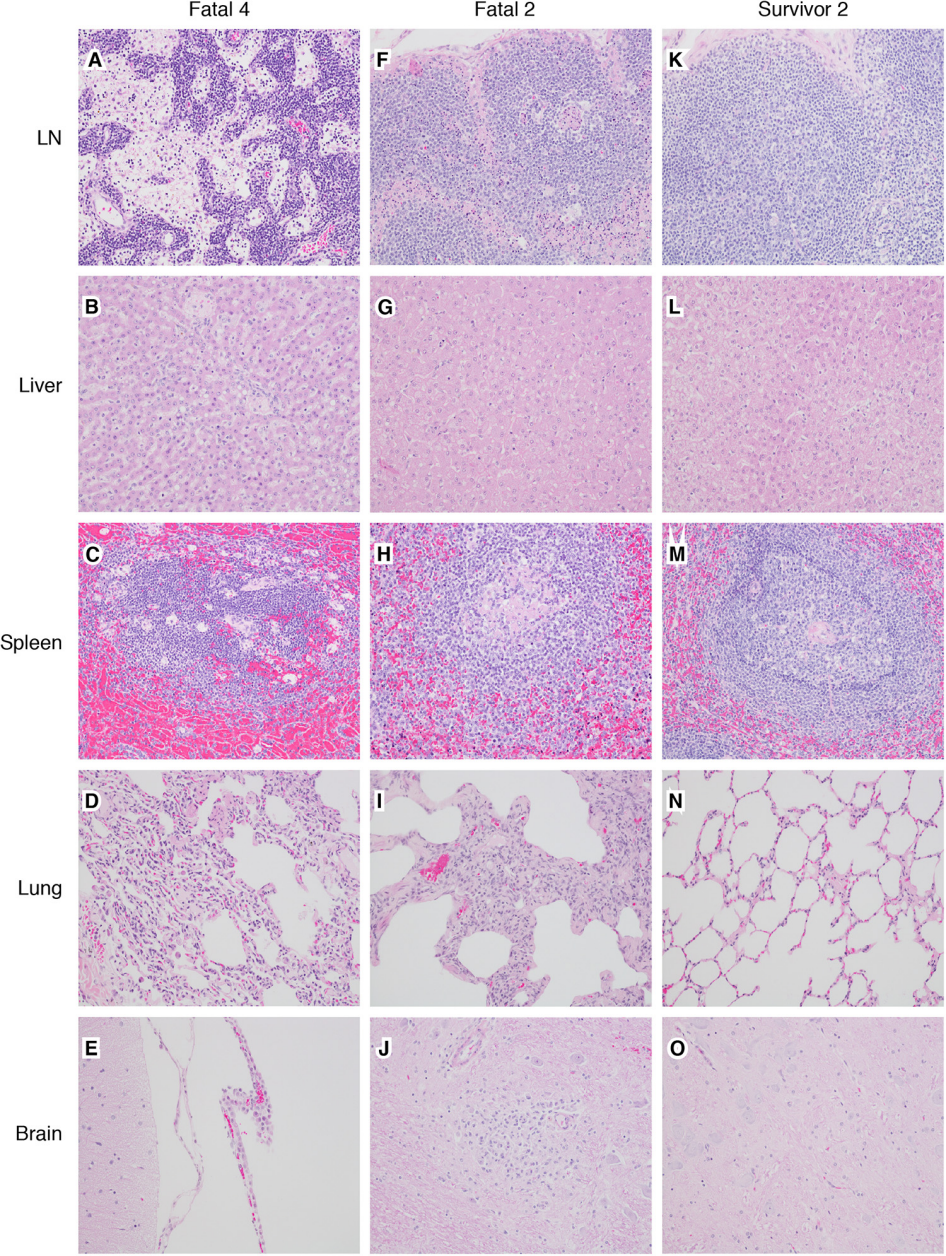
Representative histologic lesions among BDBV-infected macaques. (A to E) Fatal 4 (animal identifier). (A) Expansion of medullary sinuses in lymph node (LN) and medullary histiocytosis. (B) Expansion of hepatic sinusoidal spaces with Kupffer cell hypertrophy and hyperplasia. (C) Numerous tingible body macrophages and loss of a defined marginal zone in splenic white pulp. (D) Expansion of alveolar septa with mixed inflammatory cells and increased numbers of alveolar macrophages. (E) Modest expansion of the choroid plexus mononuclear cells. (F to J) Fatal 2. (F) Expansion of medullary and subcapsular sinuses in lymph node and histiocytosis. (G) Modest expansion of hepatic sinusoidal spaces with mixed inflammatory cells and sinusoidal leukocytosis. (H) Loss of a defined marginal zone in splenic white pulp. (I) Extensive expansion of alveolar septa with mixed inflammatory cells. (J) Well-defined glial nodule within the brainstem. (K to O) Survivor 2. (K) No significant lesions (NSL) in lymph node. (L) NSL in liver. (M) NSL in spleen. (N) NSL in lung. (O) NSL in brainstem. All images were captured at 20× magnification; tissue samples were stained with hematoxylin and eosin.

**FIG 3:**
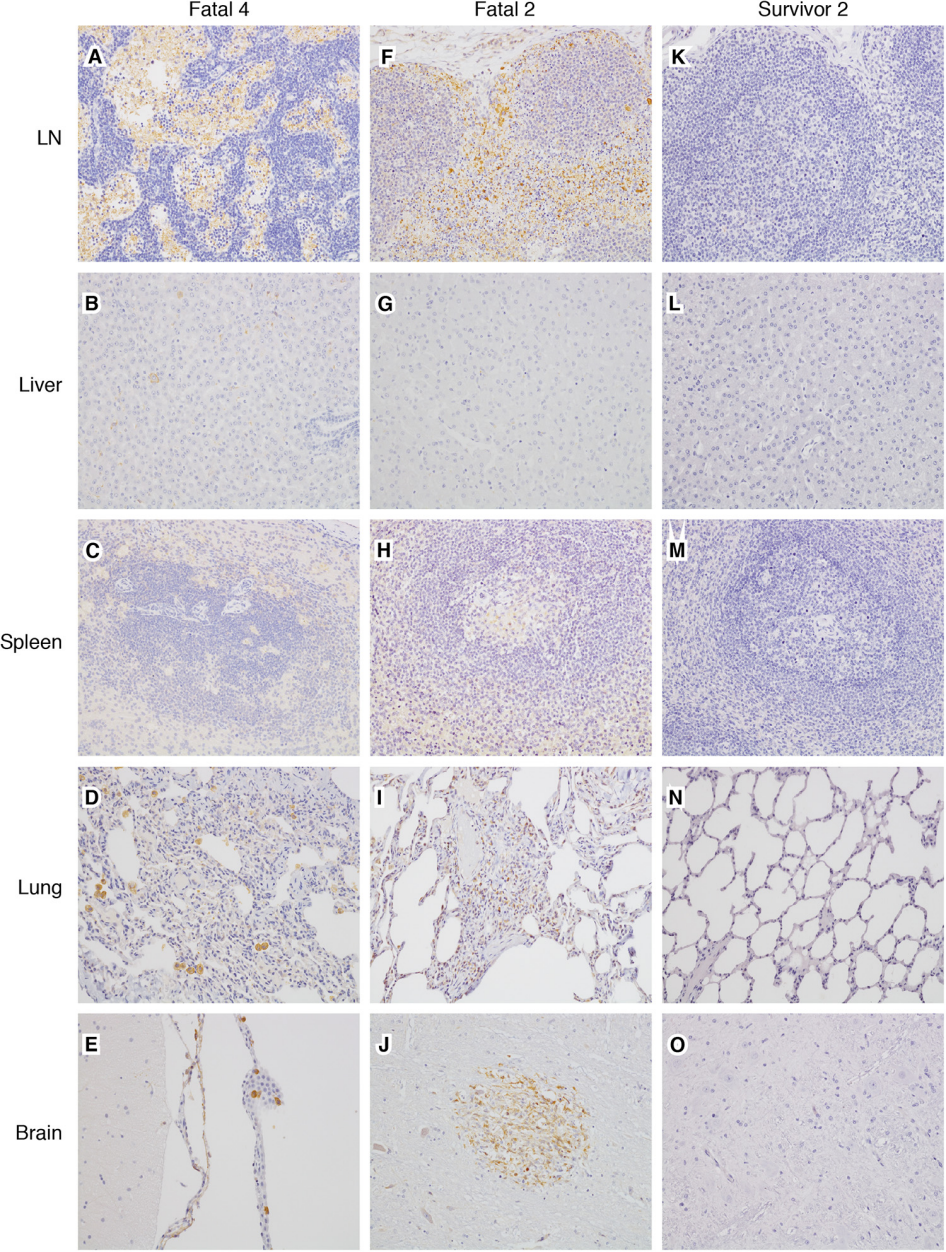
Representative immunohistochemistry (IHC) for anti-BDVD antigen in lesions among BDBV-infected macaques. (A to E) Fatal 4 (animal identifier). (A) IHC-positive histiocytes within the expanded sinuses of a lymph node. (B) Rare IHC-positive hepatocytes and scattered IHC-positive Kupffer cells. (C) IHC-positive macrophages throughout the splenic white pulp. (D) IHC-positive alveolar macrophages and mononuclear cells within the alveolar septa. (E) Scattered IHC-positive ependymal cells of the choroid plexus. (F to J) Fatal 2. (F) IHC-positive histiocytes within the expanded sinuses of a lymph node. (G) Rare IHC-positive Kupffer cells. (H) IHC-positive macrophages throughout the splenic white pulp. (I) IHC-positive mononuclear cells within the alveolar septa. (J) Focal IHC-positive glial nodule within the brainstem. (K to O) Survivor 2. (K) No significant immunolabeling (NSI) in lymph node. (L) NSI in liver. (M) NSI in spleen. (N) NSI in lung. (O) NSI in brainstem. All images captured at 20× magnification; IHC-positive cells are brown.

Macaques within the fatal cohort displayed at least one or more histologic lesions consistent with ebolavirus disease (EVD), including histiocytosis and lymphocytolysis within multiple lymph nodes (axillary and inguinal), multifocal necrotizing hepatitis, lymphohistocytic interstitial nephritis, and hemorrhagic necrotizing adrenalitis (Fig. 2). Other features included lymphohistocytic interstitial pneumonia and lymphocytic perivascular cuffs with multifocal glial nodules within the brain. Diffuse cytoplasmic immunohistochemistry (IHC) labeling for anti-BDBV GP antigen was noted in association with the aforementioned lesions (Fig. 3). IHC-positive cells included mononuclear cells scattered within the following regions: sinuses of the lymph nodes (axillary and inguinal), sinusoids of the liver (Kupffer cells), renal interstitium, adrenal medulla, alveolar septate, and alveoli of the lung (alveolar macrophages). Infrequently, antigen-positive cells included individual hepatocytes, glomerular tufts in the kidney, clusters of cells within the zona glomerulosa of the adrenal cortex, endothelium, and cells within the glial nodules of the brain. The spleens of all macaques within the fatal cohort had diminished marginal zones with relative sparing of both the mantle zone and periarteriolar sheath (PALS). In the more severe lesions, distinct zones were indiscernible, admixed with hemorrhage and numerous tingible body macrophages. The condition of the red pulp among the fatal cohort ranged from congestion to marked fibrin deposition that infiltrated the disordered marginal zones.

Interestingly, the brainstem of one survivor (survivor 1) had a focal glial nodule and multifocal clustered lymphocytic perivascular cuffs. Associated positive IHC labeling of mononuclear cells within the nodule and ependymal cells of the choroid plexus was found. No other lesions or positive IHC labeling for BDBV antigen were observed in this survivor. The remaining five survivors (survivor 2, survivor 3, survivor 4, survivor 5, and survivor 6) lacked significant lesions or IHC labeling for BDBV antigen in the tissue sections examined.

### Targeted transcriptome profiling of BDBV-infected macaques.

To identify transcriptional correlates of protection, we compared immunonomes in fatal and survivor whole blood RNA as previously described (18, 19). Samples were analyzed for each subject at early (4 to 6 dpi), middle (10 to 11 dpi), and late (14 to 15 dpi in survivors or the terminal time point in fatal subjects) disease stages (Table S1 in the supplemental material). This normalization strategy is typical for human and nonhuman primate transcriptomic studies (9–11, 13, 16, 18) on account of longitudinal sampling discrepancies and inconsistencies in disease onset and progression of individual subjects. One animal (survivor 4) was excluded due to insufficient sample availability.

10.1128/mBio.01517-21.1 TABLE S1 Individual subject BDBV GP-specific antibody titers, actual challenge doses, and sampling time points. Download Table S1, DOCX file, 0.01 MB. Copyright © 2021 Woolsey et al. 2021 Woolsey et al. https://creativecommons.org/licenses/by/4.0/ This content is distributed under the terms of the Creative Commons Attribution 4.0 International license.

**FIG 4:**
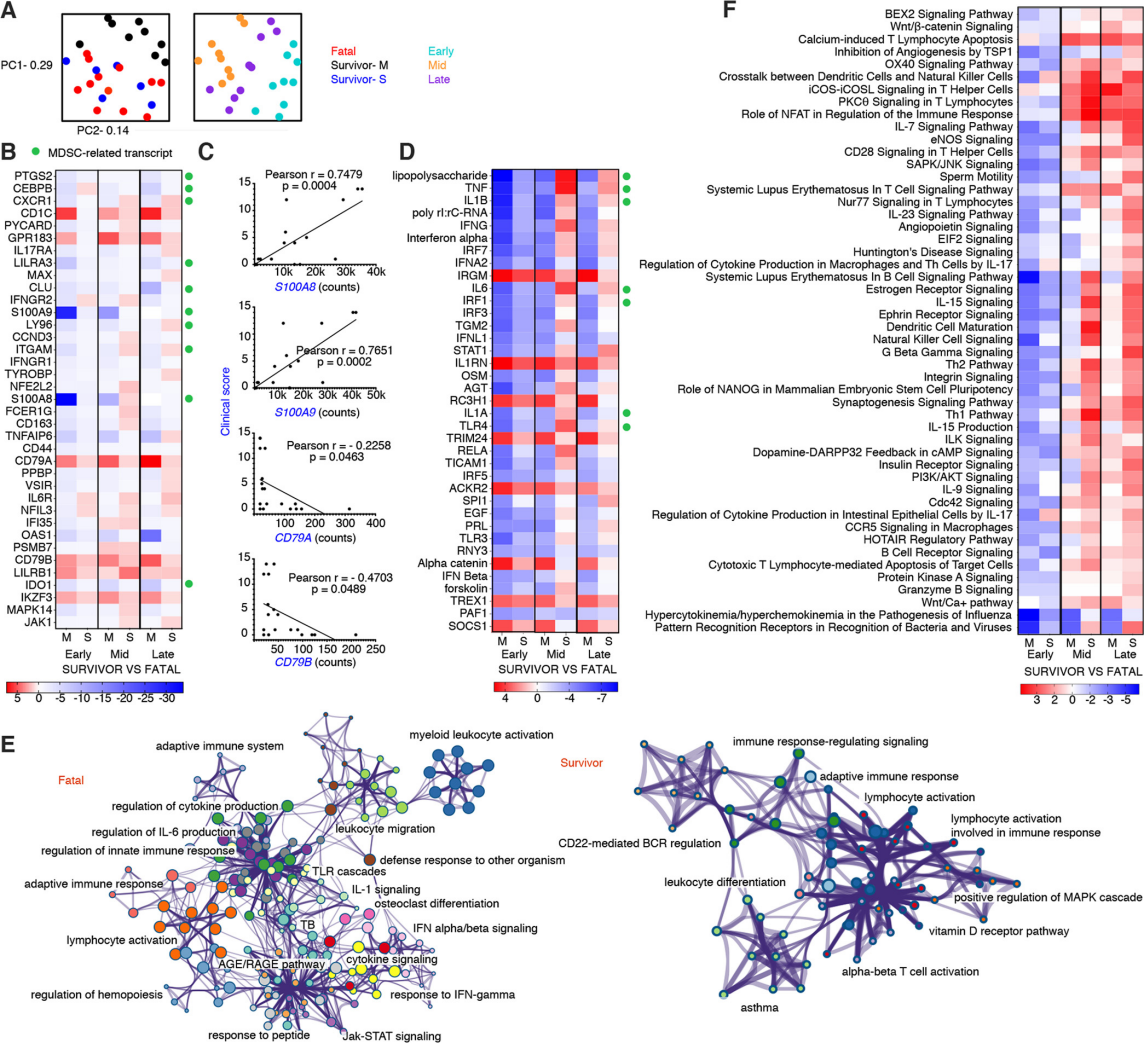
Comparison of transcriptional changes in surviving versus nonsurviving rhesus macaques infected with BDBV. (A) Shown are principal component (PC) analyses of all normalized transcripts delineated by disposition (left; fatal subjects [*n* = 4], survivors with mild-to-moderate disease [M] [*n* = 2], and survivors with severe disease [S] [*n* = 3]) and disease stage (right; early [6 to 8 dpi], middle [10 to 11 dpi], and late [13 to 19 dpi]). (B) Heatmap depicting the overall most differentially expressed transcripts in survivor versus fatal subjects. Only differentially expressed transcripts with a Benjamini-Hochberg false discovery rate (FDR)-corrected *P* value of less than 0.05 are shown. (C) Pearson correlation plots for myeloid-derived suppressor cell-related (*S100A8* and *S100A9*) and B cell receptor-affiliated (*CD79A* and *CD79B*) transcripts. (D) Heatmap of the most significantly upregulated and downregulated upstream regulators in survivor versus fatal subjects. (E) Network plots depicting gene clusters associated with BDBV infection in each fatal (left) or survivor (right) data set. Networks of enriched terms are colored by cluster identification; nodes that share the same cluster ID are typically close to each other. Terms with a *P* value of <0.01, a minimum count of 3, and an enrichment factor of >1.5 are collected and grouped into clusters based on their membership similarities. The most statistically significant term within a cluster is chosen to represent the cluster. (F) Heatmap of the most significantly upregulated and downregulated canonical pathways in survivor versus fatal subjects. Functional enrichment of all normalized transcripts at early, middle, and late stages of disease was accomplished using Ingenuity Pathway Analysis. For heatmaps, red indicates high expression, blue indicates low expression, and white indicates no difference in expression. All canonical pathways and upstream regulators had a Benjamini-Hochberg false discovery rate (FDR)-corrected *P* value of <0.05. M, survivor with mild-to-moderate disease; S, survivor with severe disease; PC1, principal component 1; PC2, principal component 2.

Examination of normalized samples by principal-component analysis (PCA) revealed that samples from fatal subjects and survivors with severe illness (subjects with a peak clinical score of ≥4) clustered together, denoting similar transcriptional profiles (Fig. 4A). Hence, survivors with severe disease were grouped separately from survivors with mild-to-moderate disease for our subsequent analyses. Dimensional separation was observed for the disease stage covariate as evidenced by distinct clustering of early-, middle-, and late-stage samples.

At the early stage of disease, we identified 98 differentially expressed (DE) transcripts with a false discovery rate (FDR)-adjusted *P* value of <0.05 in samples from survivors with mild-to-moderate versus fatal disease (see Data Set S1 in the supplemental material). The most significant DE mRNAs filtered by each disease stage are illustrated in Fig. 4B. Most notably, survivors with mild disease had an ∼30-log_2_-fold reduction in the expression of *S100A8* and *S100A9* early after infection, whereas survivors with severe disease exhibited an ∼2-log_2_-fold reduction. These mRNAs encode calcium-binding proteins that form a heterodimer (calprotectin) (20) and are considered hallmark markers of myeloid-derived suppressor cells (MDSCs) (21, 22). Repression of other MDSC-related transcripts (e.g., *PTGS2*, *CEBPB*, *CXCR1*, and *LILRA3*) along with the M2 macrophage activation marker, CD163, was also noted. Nevertheless, some of these molecules were transiently expressed in the severe disease survivor group, suggesting that disease severity may be linked to recruitment of these potent immunosuppressors. To test this hypothesis, we compared disease scores and MDSC-related transcripts. Positive correlations were found between *S100A8* (Pearson, *P* = 0.0004) and *S100A9* (Pearson, *P* = 0.0002) counts and clinical scores at middle and late disease stages (Fig. 4C).

10.1128/mBio.01517-21.1 DATA SET S1 Full list of probes detected for each sample group. Download DATA SET S1, XLSX file, 0.1 MB.. Copyright © 2021 Woolsey et al. 2021 Woolsey et al. https://creativecommons.org/licenses/by/4.0/ This content is distributed under the terms of the Creative Commons Attribution 4.0 International license.

Shared upregulated transcripts in survivors were associated with lipid antigen presentation (*CD1C*) (23), lymphocyte homing (*GPR183*) (24), B-cell receptor (BCR) signaling (*CD79B* and *CD79A*) (25), major histocompatibility complex (MHC) class I (MHC-I) inhibition (*LILRB1*) (26), and regulation of B-cell differentiation, proliferation, and maturation to an effector state (*IKZF3*) (27) (Fig. 4B). Inverse correlations were found between clinical scores and BCR-affiliated *CD79A* (Pearson, *P* < 0.05) and *CD79B* (Pearson, *P* < 0.05) transcript abundance (Fig. 4C). Thus, survival was dependent on early activation of adaptive responses with more rapid and robust signaling in the mild-to-moderate versus severe disease survivor group.

To rule out whether our sampling scheme (selected early-, middle-, and late-stage disease samples) was responsible for survivor versus fatal transcriptional changes, we also analyzed all collected blood samples independent of dpi (i.e., every collected blood sample rather than selected samples at each disease stage) (Table S1). The survivor data set still showed evidence of activation of adaptive immunity (*CD79A*, *CD79B*, *TBX21*, and HLA-related transcripts), along with reduced MDSC signaling (*S100A8*, *S100A9*, and *LILRA3*), proving that our normalization method was satisfactory (Fig. S1).

10.1128/mBio.01517-21.2 FIG S1 Volcano plot depicting overall RNA expression changes in survivor versus fatal samples irrespective of sampling time point. Download FIG S1, DOCX file, 0.4 MB. Copyright © 2021 Woolsey et al. 2021 Woolsey et al. https://creativecommons.org/licenses/by/4.0/ This content is distributed under the terms of the Creative Commons Attribution 4.0 International license.

Using the upstream analysis function of Ingenuity Pathway Analysis (IPA), we next identified DE transcriptional regulators in survivor versus fatal data sets. Survivors showed higher activation of molecules associated with autophagy regulation (*IRGM*) (28), interleukin-1 (IL-1) receptor antagonism (*IL1RN*) (29), and antiviral immunity (*TRIM24*) (30) (Fig. 4D). At the early disease stage, inhibition of lipopolysaccharide (LPS), *TNF*, and *IL1B* was projected in both survivor groups, all of which are associated with MDSC activity (21). In middle- to late-stage disease, these molecules were expressed at higher levels in the severe survivor cohort than in the fatal group but remained repressed in the mild survivor group.

To determine canonical pathways associated with protection, we performed Metascape-based functional enrichment of upregulated transcripts (FDR-adjusted *P* value of <0.05) for fatal and combined mild and severe survivor cohorts (31). Metascape/Cytoscape enables visualization of network data with the density of gene cluster interactions and nodes representing signaling intensity. Survivor signatures predominantly enriched to adaptive immunity networks, including lymphocyte activation, BCR regulation, and alpha-beta T cell activation (Fig. 4E). In the fatal cohort, transcripts primarily enriched to gene clusters involved in innate immunity, such as myeloid cell activation, leukocyte migration, Toll-like receptor (TLR) cascades, and IL-1 signaling. In contrast, adaptive immunity-related nodes were minimally apparent. To validate these findings, we also performed functional enrichment using IPA, which takes both positive and negative regulators into account for predicting activation or inhibition of each gene-signaling network. In line with our DE and Metascape results, positive z-scores in survivors correlated with adaptive immunity signatures, including ICOS signaling in T helper cells, NFAT immune regulation, calcium-induced T lymphocyte apoptosis, protein kinase C-theta (PKCθ) signaling, and CD28 signaling in T helper (Th) cells (Fig. 4F). Pathways with negative z-scores included those related to hypercytokinemia/hyperchemokinemia, pattern recognition receptor sensing, NK cell signaling, and autoimmunity (e.g., systemic lupus erythematosus [SLE] in T/B cell signaling pathways). Together, these results suggest that BDBV lethality is associated with prolonged innate signaling and minimal or dysregulated adaptive responses.

**FIG 5:**
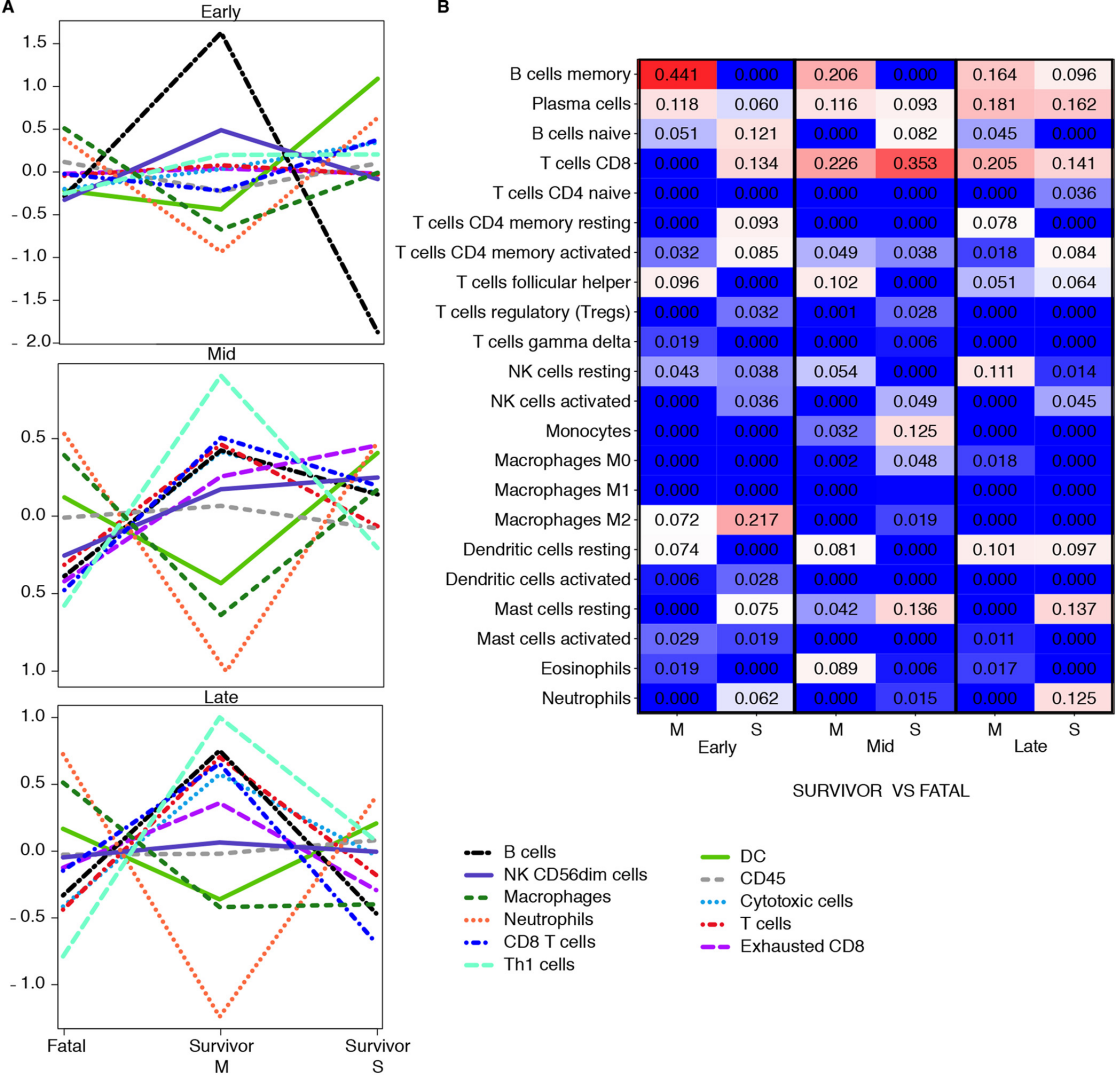
Immune cell type profiling of survivor and fatal samples. (A) Overall respective cell type quantities for each disease stage and data set (M, mild-to-moderate disease survivor [*n* = 3]; S, severe disease survivor [*n* = 2]) compared to the fatal group (*n* = 4), determined using the NanoString nSolver Advanced Analysis plugin. (B) Comparative heatmap of predicted immune cell type frequencies in each group at early, middle, and late stages of disease using CIBERSORT deconvolution software. The algorithm infers a relative increase (red) or decrease (blue) for each cell subset.

Next, to capture shifts in circulating cell populations, we used nSolver-based immune cell type profiling (Fig. 5A). In agreement with our DE analysis and enrichment results, this feature predicted that DE mRNAs in survivors with mild disease were associated with increased quantities of T helper cell, cytotoxic T cell, and B cell types. Frequencies of B cells early after infection were estimated to wane in survivors exhibiting severe disease. Neutrophil and macrophage populations were predicted to expand in fatal cases, supporting our hematology and histopathology results.

For a more granular assessment, we also performed digital cell quantification (DCQ) using CIBERSORT to estimate cell type abundances of various B and T cell subsets (Fig. 5B) (32). At the early disease stage, memory B cells and follicular helper T cells were increased in the mild survivor group, whereas CD8 T cells and memory T cells were increased in the severe survivor group. At middle and late disease stages, both survivor groups had higher quantities of plasma cell and CD8 T cell types than the fatal cohort. These data suggest that both humoral and cellular responses are critical for survival against BDBV.

### Assessment of humoral responses.

**FIG 6:**
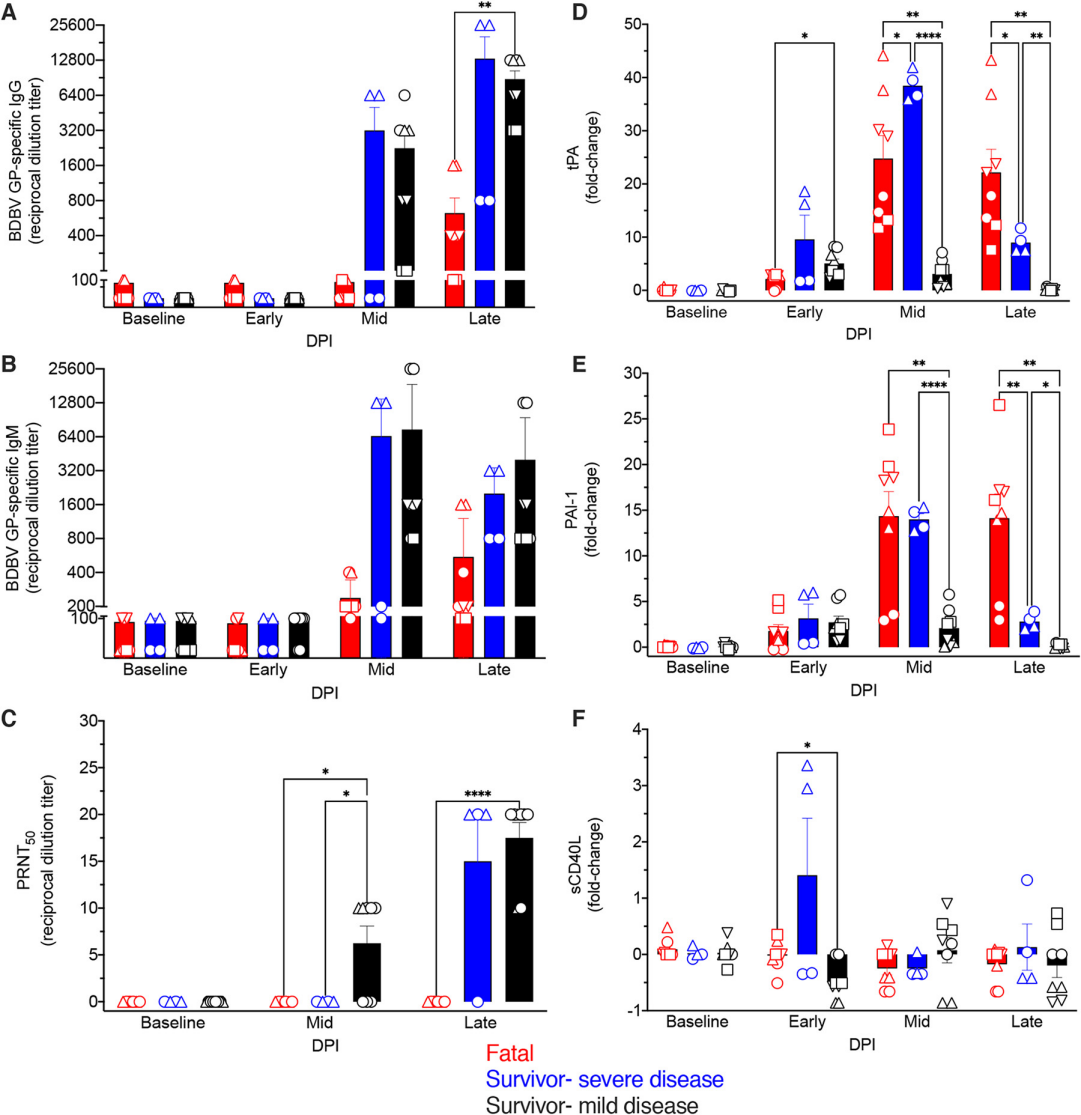
Antibody titers and levels of thrombosis-associated markers in BDBV-infected macaques. (A and B) BDBV glycoprotein-specific immunoglobulin G (IgG) (A) and immunoglobulin M (IgM) (B) titers in serum samples of fatal (*n* = 4) and survivor (*n* = 6 [*n* = 4 with mild-to-moderate disease and *n* = 2 with severe disease]) subjects were measured at early, middle, and late stages of disease. (C) Neutralizing antibody titers in BDBV-infected macaques determined by plaque reduction neutralization tests. (D to F) Fold change increases or decreases in thrombosis-associated markers in BDBV-infected macaques for each group. Each replicate is shown with symbols denoting data for individual subjects (*n* = 10 biologically independent animals/samples in a single experiment); each bar and error bar represents the group mean value ± SEM. Statistical significance was determined using two-way ANOVA with Greenhouse-Geisser correction. *, *P* < 0.05; **, *P* < 0.001; ***, *P* < 0.0001; ****, *P* < 0.00001.

As our DCQ predictions suggested that survival correlated with recruitment of plasma cells, we performed antiglycoprotein (anti-GP) IgG (Fig. 6A) and IgM (Fig. 6B) enzyme-linked immunosorbent assays (ELISAs) on serum samples collected from each subject. Survivors formed BDBV GP-specific IgM and IgG, with both immunoglobulin classes appearing at the middle stage of disease. At the late stage of disease, the antibody titers of survivors with severe disease ranged from 1:800 to 1:25,600 for IgG and 1:800 to 1:3,200 for IgM, whereas the titers in survivors with mild disease ranged from 1:3,200 to 1:12,800 for IgG and 1:800 to 1:12,800 for IgM. Conversely, only low IgM and IgG (1:100 to 1:1,600) titers were noted in fatal subjects. For the surviving subject (survivor 5) with only viral RNA detectable and not infectious virus loads, we observed moderate to high IgM and IgG titers (Table S1). Unlike the immunoglobulin levels in the fatal group, the IgM titers in the survivor cohorts generally declined during the late disease stage, conjointly with increasing moderate to high titers of IgG (Fig. 6A and B). Plaque reduction neutralization tests indicated that survivors had higher, albeit overall low levels of neutralizing antibodies at middle- and late-stage disease (Fig. 6C).

### Measurement of thrombosis-associated markers.

As EVD is known to induce disseminated intravascular coagulation, the concentrations of various thrombosis-associated markers were measured using a cytokine bead array at each disease stage (4). Elevated levels of tissue plasminogen activator (tPA) (Fig. 6D) and plasminogen activator inhibitor 1 (PAI-1) (Fig. 6E) were found in fatal macaques and survivors with severe disease at middle and late disease stages, although declines in these markers were seen in the severe survivor cohort at the latter time point. Interestingly, tPA and soluble CD40 ligand (sCD40L) levels were significantly different in the mild survivor group only at the early disease stage (Fig. 6D and F). These results indicate a prompt return to coagulation homeostasis in these subjects.

### Plasma cytokine bead array analysis.

**FIG 7:**
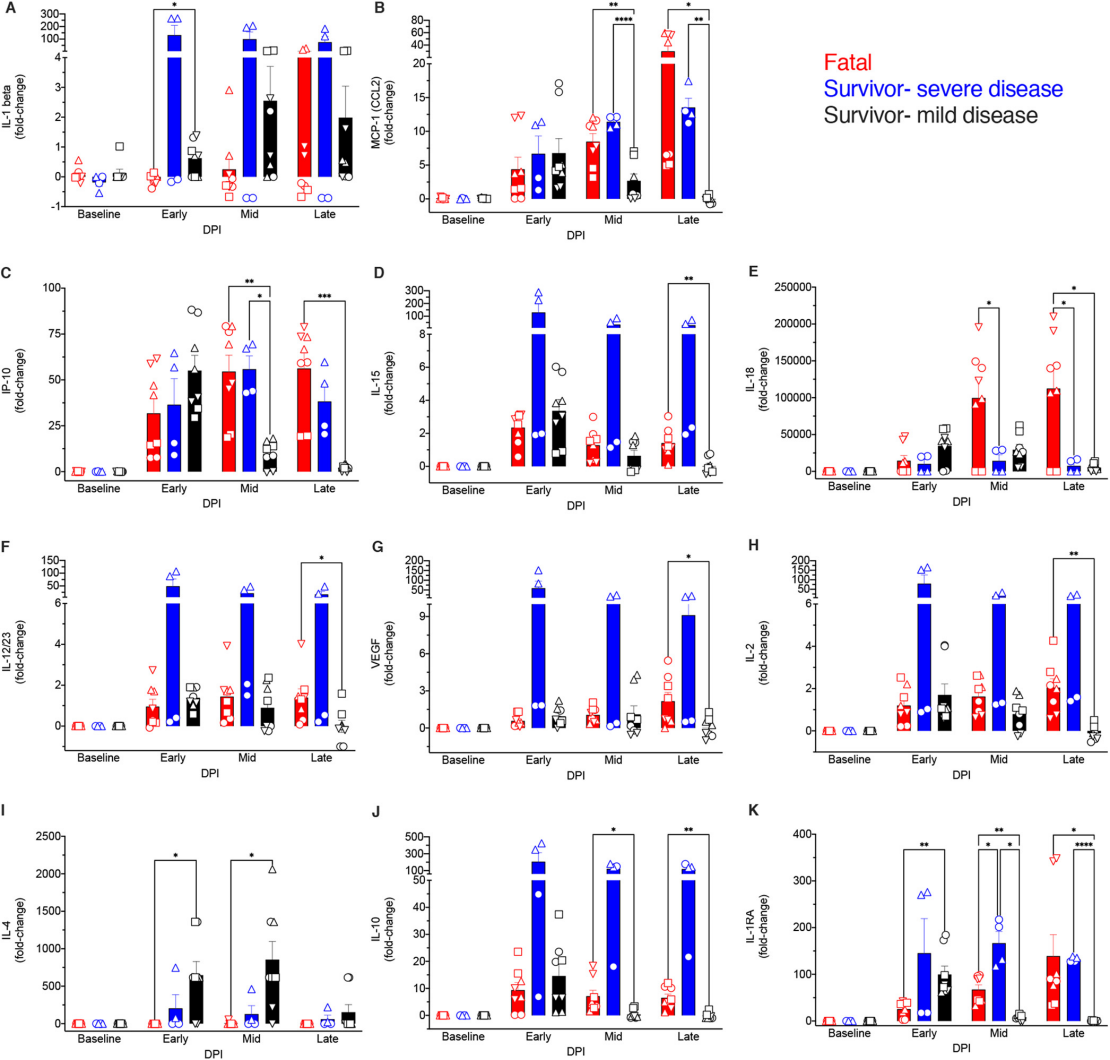
Comparison of plasma cytokine/chemokine levels in BDBV-infected rhesus macaques. (A to K) Fold change increases or decreases in selected cytokines, chemokines, or other soluble mediators grouped by disposition (*n* = 4 fatal and *n* = 6 survivor [*n* = 4 with mild-to-moderate disease and *n* = 2 with severe disease] subjects) and disease stage. Each replicate is shown with symbols denoting data for individual subjects (*n* = 10 biologically independent animals/samples in a single experiment); each bar and error bar represents the group mean value ± SEM. Statistical significance was determined using two-way ANOVA with Greenhouse-Geisser correction. *, *P* < 0.05; **, *P* < 0.001; ***, *P* < 0.0001; ****, *P* < 0.00001.

Finally, soluble mediators (circulating cytokines, chemokines, and growth factors) were measured in BDBV-infected macaque plasma samples. IL-1β levels were higher in mild disease survivors early after infection, suggesting that innate immunity was briefly stimulated and then arrested in this cohort (Fig. 7A). Severe disease and lethality were instead associated with copious increases in markers of innate immune function and inflammation that persisted late into disease, e.g., monocyte chemoattractant protein 1 (MCP-1), IP-10, IL-15, IL-18, IL-12/23, and vascular endothelial growth factor (VEGF) (Fig. 7B to E) (33). The expression of these proinflammatory mediators coincided with the onset of clinical disease, similar to previous EBOV reports. Higher systemic increases in IL-2 and anti-inflammatory (IL-10 and IL-1 receptor antagonist [IL-1RA]) mediators were also observed in fatal subjects. The Th2 cytokine IL-4 was more abundantly expressed in survivors in early- to middle-stage disease, which coincides with our enrichment findings indicating activation of both Th2 and Th1 pathways. Additionally, IL-1RA was expressed at higher levels in survivors at the early disease stage. These results indicate that rapid resolution of inflammation was accomplished in survivors with mild disease.

## DISCUSSION

The continued reemergence of ebolaviruses emphasizes the need for the development of effective countermeasures against these viruses (2–4). Historically, efforts to develop interventions have been hampered, in part, by the dearth of clinical and laboratory research exploring the pathophysiology of EVD. Moreover, clinical samples typically provide only glimpses into the host response, as repeated sampling is uncommon. Prognostic indicators comprising factors that promote or hinder defense against ebolaviruses, particularly BDBV, thus remain incompletely defined. The reduced lethality of BDBV in NHP models (6–8), resulting in a pool of survivors, afforded us the unique opportunity of identifying immune correlates that confer protection against ebolaviruses. NHPs are considered the gold standard animal model, as they most accurately recapitulate human EVD (5).

We compared viral loads, clinical signs, and host responses in BDBV-infected rhesus macaques. Surprisingly, there was no significant difference between peak viral RNA titers in fatal and survivor macaques, but there was a significant difference in infectious titers. This finding may reflect comparable initial dissemination of the virus in each cohort but the inability to remove infected cells and terminate the immune response in fatal cases. Immunological control, whether mediated by innate or adaptive responses, is thus likely responsible for infectious virus clearance in survivors. All animals displayed various degrees of illness along with hematological changes. Disease severity and fatal outcome corresponded with liver dysfunction, lymphopenia, thrombocytopenia, and coagulopathy.

Disseminated intravascular coagulation (DIC) is a hallmark feature of EVD and is characterized by hyperactivation of the coagulation cascade (4). Consequences of DIC include hemorrhagic diathesis due to consumption of platelets and clotting factors, as well as widespread deposition of fibrin clots (thrombi) that become trapped in small blood vessels, resulting in ischemia, hemolytic anemia (erythrocytes are fragmented during transport through narrowed microvasculature), and occasionally organ failure. One key event that triggers this EVD phenomenon is oversecretion of tissue factor by monocytes and macrophages (34). Another mechanism involves increased levels of plasminogen activator inhibitor I (PAI-I) in response to marked increases in cytokines and/or circulating LPS (35). The thrombosis-associated markers PAI-1 and tissue plasminogen activator (tPA) were elevated in fatal macaques and survivors exhibiting severe illness after BDBV exposure, suggesting that these factors may also play a role in DIC. Higher PAI-1 levels were also found in pediatric patients infected with *Sudan ebolavirus* (36) and fatal human cases of Lassa virus disease (37), suggesting that this marker may represent a universal feature among hemorrhagic fever virus infections. PAI-1 is a serine protease inhibitor (serpin) that normally functions as an inhibitor of plasminogen activators like tPA and urokinase (38). In that sense, PAI-1 halts, whereas tPA promotes, fibrinolysis. While both PAI-1 and tPA were elevated among fatal macaques and survivors with severe disease, exceptionally high levels of tPA may enhance spontaneous fibrinolysis, alluding to the complexity of fibrinolysis homeostasis. Coagulation changes that mimic DIC may also play a role in the severity of coronavirus disease 2019 (COVID-19). A recent paper revealed that elevation of tPA and PAI-1 was significantly associated with mortality in COVID-19 patients (39). A strong correlation was found between tPA/PAI-1 concentrations and both absolute neutrophil counts and systemic calprotectin (S100A8/S100A9 heterodimer) levels.

Our results indicated that severe and fatal disease also corresponded with granulocytosis and monocytosis. Neutrophil granulocytes are spared from EBOV infection, but their degranulation, proinflammatory secretion, neutrophil extracellular trap (NET) formation, and reactive oxygen species (ROS) production can contribute to inflammation and tissue damage (40). *In vitro* and *in vivo*, EBOV readily infects monocytes and macrophages, leading to their activation and resulting in massive cytokine, chemokine, and growth factor secretion (41–44). Our studies and others have shown that monocytes are the major cell targets for EBOV *in vivo* and constitute a significant proportion of transcriptional changes in blood cells from infected macaques (12, 16). These studies support the notion that monocytes and/or neutrophils are strongly implicated in EVD pathophysiology.

Our analyses indicated that a fatal outcome was associated with MDSC-related transcriptional signatures (e.g., *S100A8*, *S100A9*, *PTGS2*, *CEBPB*, *LILRA3*, and *CXCR1*) (21). MDSCs are pathologically activated monocytes and neutrophils with potent immunosuppressive activity (22). The morphological and physiological resemblance of MDSCs to conventional monocytes and granulocytes makes it difficult to distinguish between these cell types. To further complicate matters, based on their theorized granulocytic or monocytic lineage, two MDSC subsets exist, granulocytic/polymorphonuclear MDSC (PMN-MDSC) and monocytic MDSC (M-MDSC), each with distinct cellular profiles and mechanisms of immunosuppression. Whereas classical activation of myeloid cells is driven primarily by pattern recognition receptors (PRRs) and quickly subsides after clearance of the stimulus, pathological activation arises from a persistent environment of growth factors (granulocyte-macrophage colony-stimulating factor [GM-CSF] and macrophage colony-stimulating factor [M-CSF]), chemokines (CCL2 [MCP-1]), and inflammatory signals (IL-1 beta, IL-18, VEGF, IL-6, HIF1-alpha, and adenosine) in the absence of pathogen clearance (21, 22, 45, 46). In the present study, the expression of many of these polarizing factors was associated with disease severity or fatal outcome following BDBV exposure. Great effort has been put forth in recent years to define the genomic, proteomic, and metabolic signatures of MDSCs, given their implication in numerous pathological conditions, including sepsis, cancer, chronic infections, and various autoimmune disorders (e.g., type 1 diabetes, rheumatoid arthritis, and systemic lupus erythematosus [SLE]) (21, 47, 48). Our results showed increased transcripts mapping to SLE in T/B cell signaling pathways in the fatal data set, implying that EVD and SLE may share certain pathological features.

Other supporting evidence for the role of MDSC in filovirus pathogenesis is reports of an accumulation of low-density neutrophils in humans or NHPs infected with EBOV (9, 16, 49). PMN-MDSCs have a lower density than conventional neutrophils, enabling them to fractionate with the peripheral blood mononuclear (PBMC) interface in density gradient blood preparations. Moreover, our group and others have identified rapidly expanding monocyte-like populations that express low levels of MHC-II molecules, a defining feature of M-MDSCs (12, 50, 51). One caveat of our study is that we did not perform flow cytometry or single-cell RNA sequencing (scRNA-Seq) to determine whether MDSCs were indeed recruited and contributed to the bulk DE transcripts identified. However, our previous transcriptional analysis of purified monocytes from infected macaques supports monocytes as major contributors of gene expression changes in peripheral cells that express high levels of MDSC-related transcripts, including many that we have identified in this study (12). More recently, comprehensive scRNA-Seq, along with high-dimensional flow cytometry, confirmed that monocyte-like cells from EBOV-infected rhesus macaques express high levels of *S100A8*/*S100A9* and downregulate multiple MHC-II-related molecules (16). The authors of this study suggested these cells might represent immature monocytes or bone marrow monocyte precursors released during emergency myelopoiesis. Importantly, future studies should test whether these cells represent *bona fide* MDSCs by assessing their ability to suppress the adaptive immune response via *ex vivo* functional assays. M-MDSCs can suppress T cell activity by secreting nitric oxide (NO) and immunosuppressive cytokines (IL-10 and transforming growth factor beta [TGF-β]) or expressing immune regulatory molecules (PDL-1). PMN-MDSCs preferentially exploit ROS, prostaglandins, peroxynitrite, and arginase 1 to mediate immune suppression (21).

Macrophages that are differentiated from M-MDSCs but not monocytes are immunosuppressive and share similar genomic profiles. Kwak et al. demonstrated that the immunosuppressive activity of M-MDSC-derived macrophages is dependent on prolonged expression of S100A9 protein in these cells and involves the transcription factor C/EBPβ (52). The authors also demonstrated that S100A9 promotes M2 polarization of macrophages. In contrast to M1 macrophages, which participate in pathogen killing, M2 alternatively activated macrophages are generally anti-inflammatory. Therefore, these cells could promote an environment that enhances viral replication or downregulates the adaptive immune response. McElroy et al. showed substantial immunoreactivity of the M2-affiliated marker CD163 in association with viral antigen in the tissues of human fatal cases (53). This marker was associated with both disease severity and fatal outcome. Our results also indicated higher expression of *CD163* in fatal subjects, as well as abundant tingible body macrophages in lymphoid tissue, further supporting that M2-like macrophages contribute to ebolavirus pathogenesis. Tingible body macrophages are thought to downregulate the germinal center reaction by releasing prostaglandins and inhibiting IL-2 production, which may contribute to the lack of adaptive responses (54). Disruption of the B cell-rich marginal zone within the germinal center architecture in lymphoid tissue may exacerbate this condition, as this finding was prominent in fatal cases in our study.

Conversely, germinal centers were intact in surviving animals with the absence of an accumulation of tingible body macrophages; analysis of DE transcripts demonstrated that survivors also expressed more transcripts enriching to B cell antigen receptor signaling (*CD79B* and *CD79A*) (25). Previous studies have shown that downregulation of *CD79A* is observed in B cells during acute disease following exposure of rhesus macaques to EBOV (16), which may serve as a virus mechanism to impede B cell activation and generation of memory B cells. Indeed, lower predicted frequencies of IgM and IgG memory B cells, neutralizing titers, and transcriptionally derived plasma cell quantities were detected in fatal macaques. In contrast, estimated increases in these B cell subsets were observed in survivors, along with higher ELISA titers of BDBV-specific IgM and IgG antibodies and neutralizing titers. Transcripts mapping to numerous cellular immunity- and humoral immunity-related pathways, including ICOS signaling in T helper cells (55), calcium-induced T lymphocyte apoptosis (56), PKCθ signaling (57), and CD28 signaling in T helper (Th) cells (58), were also higher in survivor than in fatal data sets. Although lymphocyte activation signaling was found in fatal cases, these transcriptional responses appeared nonspecific rather than a result of antigen-dependent T and B cell activation and were possibly cytokine mediated (16). Therefore, both antigen-specific humoral and cellular immunity are likely pivotal for protection against EVD.

In summary, we identified potential biomarkers that predict EVD disease severity and lethality. Sustained activation of innate immunity, MDSC-related signaling, and dysregulation of fibrinolytic pathways were prominent findings in fatal cases. Survivors expressed T cell- and B cell-related transcripts and other mRNAs mapping to adaptive immune pathways, signifying that both cellular and humoral immunity are critical for protection against EVD. Elucidation of the mechanisms that confer lethality or defense against ebolaviruses can be harnessed to develop diagnostics or immunomodulatory therapies for these deadly pathogens.

## MATERIALS AND METHODS

### Ethics statement.

Animal studies were performed in biosafety level 4 (BSL4) biocontainment at the University of Texas Medical Branch (UTMB) and approved by the UTMB Institutional Biosafety Committee. Animal research was conducted in compliance with the UTMB IACUC, the Animal Welfare Act (59), and other federal statutes and regulations relating to animals. The UTMB animal research facility is fully accredited by the Association for Assessment and Accreditation of Laboratory Animal Care.

### Challenge virus.

BDBV (strain 200706291; GenBank accession no. MK028856.1) was isolated from a fatal human case in western Uganda during the 2007 outbreak (46). The challenge stock used in this study was kindly provided by Thomas G. Ksiazek and was propagated on Vero E6 cells twice (passage 2 virus). Stocks were certified free of endotoxin and mycoplasma contamination.

### Animal infection.

Ten adult (5 females and 5 males) rhesus macaques (Macaca mulatta) weighing 2.64 to 6.98 kg that served as untreated controls on seven different studies at the Galveston National Laboratory were employed for this project. All macaques (source PrimGen) were i.m. challenged in the left quadriceps with a 1,000-PFU target dose (actual dose was 750 to 1,088 PFU) of the same BDBV challenge stock (Table S1). An internal scoring protocol was implemented to track disease progression in challenged animals and included criteria like behavior, posture and activity level, appetite, respiration, and the presence of hemorrhagic manifestations, as described previously (8, 11, 12, 14). Animals were checked at least twice daily, and subjects that reached a clinical score of ≥9 were euthanized with a pentobarbital solution. Longitudinal blood samples were taken over the course of the study, and tissue samples from major organs were taken at the time of euthanasia (Table S1). All measurements requiring physical manipulation were performed under ketamine sedation.

### Clinical pathology.

EDTA-treated blood was analyzed using a laser-based Beckman Coulter Ac-T diff hematology analyzer to determine total white blood cell counts, white blood cell differentials, red blood cell counts, platelet counts, hematocrit values, total hemoglobin concentrations, mean cell volumes, mean corpuscular volumes, and mean corpuscular hemoglobin concentrations. A Piccolo point-of-care analyzer and biochemistry panel plus analyzer discs (Abaxis) were used to test for serum concentrations of albumin, amylase, alanine aminotransferase (ALT), aspartate aminotransferase (AST), alkaline phosphatase (ALP), gamma-glutamyltransferase (GGT), glucose, cholesterol, total protein, blood urea nitrogen (BUN), creatinine (CRE), uric acid, and C-reactive protein (CRP).

### Histopathology and immunohistochemistry.

Necropsy was performed on all subjects in the BSL4 facility. Tissue samples for histopathologic and immunohistochemical (IHC) examination were immersed in 10% neutral buffered formalin for at least 21 days, followed by a change of formalin, before removal from the BSL4 facility. Inactivated tissue samples were processed in a BSL1 facility. Tissue sections were deparaffinized and rehydrated through xylene and graded ethanol. Slides went through heat-induced antigen retrieval in a steamer at 95°C for 20 min in Sigma citrate buffer, pH 6.0, 10× (Sigma-Aldrich, St. Louis, MO). To block endogenous peroxidase activity, slides were treated with 3% hydrogen peroxide and rinsed in distilled water. The tissue sections were processed for IHC using the Thermo Autostainer 360 (ThermoFisher, Kalamazoo, MI). Sequential 15-minute incubations with avidin D and biotin solutions (Vector, Burlingame, CA) were performed to block endogenous biotin reactivity. Specific anti-BDBV immunoreactivity was detected using an anti-BDBV GP primary antibody at a 1:2,000 dilution for 60 min (IBTS Services, Gaithersburg, MD). Secondary biotinylated goat anti-rabbit IgG antibody (BA-1000; Vector Laboratories, Burlingame, CA) was added at a dilution of 1:200 and incubated for 30 min. Next, Vector horseradish peroxidase streptavidin, ready-to-use (RTU) (Vector) was added for an additional 30 min. Slides were developed with Dako DAB (diaminobenzidine) chromogen (K3468; Dako, Carpinteria, CA) for 5 min and counterstained with hematoxylin for 30 s.

### RNA isolation.

On the specified procedure days, blood was collected from each macaque by femoral venipuncture into BD Vacutainer EDTA tubes (BD Biosciences, San Jose, CA). An aliquot of EDTA-treated whole blood (100 μl) was diluted with 600 μl of buffer AVL inactivation buffer (Qiagen, Hilden, Germany), and RNA was extracted using a viral RNA minikit (Qiagen) according to the manufacturer’s instructions.

### Viral-load determination.

OneStep probe RT-qPCR kits (Qiagen) and the CFX96 system and software (Bio-Rad) were used to determine BDBV viral copies. To detect viral RNA, we targeted the BDBV VP35 intergenic region or the GP gene with primer pairs and a 6FAM (6-carboxyfluorescein)-5′-AGGCTTCCCTCGCTGCCGTTATG-3′-TAMRA (6-carboxytetramethylrhodamine) or a 6FAM-CGCAACCTCCACAGTCGCCT-TAMRA probe, respectively. Thermocycler run settings were 50°C for 10 min; 95°C for 10 s; and 40 cycles of 95°C for 10 s plus 57°C (59°C for GP) for 30 s. Integrated DNA Technologies synthesized all primers, and Life Technologies customized the probes. Representative BDBV genomes were calculated using a genome equivalent standard, which takes into account Avogadro’s number and the molecular weight of the BDBV genome. The limit of detection for this assay is 1,000 copies/ml.

The titers of infectious virus loads were determined using a standard plaque assay and Vero E6 cells (catalog number CRL-1586; ATCC). Briefly, increasing 10-fold dilutions of plasma samples were adsorbed to Vero E6 monolayers in duplicate wells (200 μl), overlaid with 0.8% agarose/2× Eagle minimal essential medium (EMEM), and incubated for 6 days at 37°C in 5% CO_2_. Neutral red stain was added, and plaques were counted after a 24- to 48-h incubation. The limit of detection for this assay is 25 PFU/ml.

### NanoString sample preparation.

NHPV2_Immunology reporter and capture probe sets (NanoString Technologies) were hybridized with 5 μl of each RNA sample at 65°C for at least 12 h. The RNA-probe set complexes were then loaded into an nCounter microfluidics cartridge and assayed on a NanoString nCounter SPRINT Profiler. To estimate the abundance of each of the 769 unique mRNA targets included in the NHPV2_Immunology panel, fluorescent reporter barcodes were imaged and counted in each sample lane. To meet quality control (QC) criteria, samples with an image binding density greater than 2.0 were reanalyzed with 2 μl of RNA. NanoString barcoding technology was previously validated for EVD gene expression (11). The RNA was hybridized with NanoString NHPV2_Immunology reporter and capture probe sets, and the RNA-probe set complexes were loaded onto an nCounter SPRINT Profiler to determine mRNA counts. This platform enables the detection of up to 769 NHP-specific immune-related transcript targets.

### Bioinformatics analysis.

nCounter .RCC files were imported into NanoString nSolver 4.0 software. All samples met the established QC criteria. To compensate for differing RNA inputs, housekeeping genes and spiked-in positive and negative controls were used to normalize raw counts. The data were analyzed using the NanoString nSolver Advanced Analysis 2.0 package to generate principal component (PC) figures, cell type trend plots, and mRNA expression heatmaps (a full list of probes detected for each sample group, along with fold change values and *P* values, can be found in Data Set S1 in the supplemental material). Normalized data were exported as a .CSV file, and human annotations were added for each respective gene to perform immune cell profiling within nSolver. Functional enrichment of survivor versus fatal normalized counts at early-, middle-, and late-stage disease was accomplished using Ingenuity Pathway Analysis software (Qiagen). Z-scores were imported into GraphPad Prism version 9 to produce canonical signaling and upstream regulator heatmaps. To generate the network maps, DE mRNAs with an FDR-adjusted *P* value of <0.05 from each bronchoalveolar lavage (BAL) fluid sample or blood sample data set were imported into Metascape and visualized using Cytoscape (31). To validate our nSolver-derived cell type predictions, we used CIBERSORT deconvolution software (32).

### Bead-based multiplex immunoassays.

The concentrations of circulating cytokines, chemokines, and other analytes were assayed using bead-based multiplex technology. Irradiated plasma samples were incubated with magnetic beads from Milliplex NHP cytokine premixed 23-plex panel (EMD Millipore, Billerica, MA) kits according to the recommendations provided. Analytes measured included IL-1β, IL-1 receptor antagonist (IL-1RA), IL-2, IL-4, IL-5, IL-6, IL-8, IL-10, IL-12/23 (p40), IL-13, IL-15, IL-17, IL-18, gamma interferon (IFN-γ), granulocyte colony-stimulating factor (G-CSF), granulocyte-macrophage colony-stimulating factor (GM-CSF), monocyte chemoattractant protein 1 (MCP-1), macrophage inflammatory protein 1α (MIP-1α), MIP-1β, tumor necrosis factor alpha (TNF-α), transforming growth factor alpha (TGF-α), soluble CD40 ligand (sCD40L), and vascular endothelial growth factor (VEGF). The concentrations in each plasma sample were measured using a Bioplex-200 array system (Bio-Rad, Hercules, CA).

Serum concentrations of other immune mediators (IL-6, IL-10, IP-10, IL-1β, IL-12p40, IL-17A, IFN-β, IL-23, TNF-α, IFN-γ, GM-CSF, IL-8, and MCP-1) and plasma-derived thrombosis-associated markers (P-selectin, D-dimer, PSGL-1, tPA, sCD40L, PAI-1, and factor IX) were determined by flow cytometry using LegendPlex multiplex technology (BioLegend) and the nonhuman primate inflammation 13-plex (1:4 dilution) and human thrombosis (1:100 dilution) kits, respectively. Samples were processed in duplicate following the kit instructions and recommendations. Following bead staining and washing, 1,500 to 4,000 bead events were collected on a FACSCanto II cytometer (BD Biosciences) using BD FACSDiva software. The raw .fcs files were analyzed with BioLegend’s cloud-based LEGENDplex data analysis software.

### Anti-BDBV GP IgM and IgG ELISA.

Sera collected at the indicated time points were tested for BDBV GP-specific IgM and IgG antibodies by ELISA. MaxiSorp 96-well plates (catalog number 44-204; Thermo Fisher, Rochester, NY) were coated overnight with 15 ng/well (0.15 ml) of recombinant BDBV GP lacking the transmembrane region (GPΔTM; Integrated Biotherapeutics, Gaithersburg, MD) in a sodium carbonate/bicarbonate solution (pH 9.6). Antigen-adsorbed wells were subsequently blocked with 4% bovine serum antigen (BSA) in 1× phosphate-buffered saline (PBS) for at least 2 h. Sera were initially diluted 1:100 and then 2-fold through 1:12,800 in ELISA diluent (1% BSA in 1× PBS and 0.2% Tween 20). After a 1-h incubation, cells were washed six times with wash buffer (1× PBS with 0.2% Tween 20) and incubated for an hour with a 1:2,500 dilution of HRP-conjugated anti-monkey IgM or IgG (Fitzgerald Industries International, Acton, MA). SigmaFast *O*-phenylenediamine (OPD) substrate (product number P9187; Sigma) was added to the wells after six additional washes to develop the colorimetric reaction. The reaction was stopped with 3 M sulfuric acid 10 to 15 min after the addition of OPD, and absorbance values were measured at a wavelength of 492 nm on a spectrophotometer (Emax system; Molecular Devices, Sunnyvale, CA). Absorbance values were normalized by subtracting the values for uncoated wells from the values for antigen-coated wells at the corresponding serum dilution. End-point titers were defined as the reciprocal of the last adjusted serum dilution with a value of ≥0.40.

### Statistical analysis.

All statistical analyses were carried out in GraphPad Prism version 9. No data were excluded. Two-way repeated-measures analysis of variance (ANOVA) with Greenhouse-Geisser correction and Tukey’s multiple-comparison test was used to obtain *P* values for cytokine/chemokine levels, antibody titers, and thrombosis-associated markers. Peak viremia was evaluated using the Mann-Whitney nonparametric *t* test. A multiple-hypothesis Benjamini-Hochberg false discovery rate (FDR)-corrected *P* value of less than 0.05 was deemed significant for transcriptional analyses, unless otherwise stated.
