# Supplementary material for: Bundibugyo ebolavirus Survival Is Associated with Early Activation of Adaptive Immunity and Reduced Myeloid-Derived Suppressor Cell Signaling
Source: mBio. 2021 Aug 10;12(4):e01517-21. doi: 10.1128/mBio.01517-21 (PMC8406165; doi:10.1128/mBio.01517-21)
Supplement: TABLE S1 [file mbio.01517-21-st001.docx]

| **Animal ID** | **AVG IgG (reciprocal dilution titer)** | **AVG IgM (reciprocal dilution titer)** | **Sampling** | **Actual challenge dose** |
| --- | --- | --- | --- | --- |
| Fatal 1 | 0 (early), 0 (mid), 400 (late) | 0 (early), 150 (mid), 300 (late) | 0 (baseline), 3, 6 (early), 10 (mid), 14 (late) | 838 pfu |
| Fatal 2 | 100 (early), 100 (mid), 1600 (late) | 0 (early), 400 (mid), 1600 (late) | 0 (baseline), 3, 6 (early), 10 (mid), 14, 19 (late) | 838 pfu |
| Fatal 3 | 0 (early), 0 (mid), 400 (late) | 100 (early), 200 (mid), 200 (late) | 0 (baseline), 3, 6 (early), 10 (mid), 13 (late) | 838 pfu |
| Fatal 4 | 0 (early), 50 (mid), 100 (late) | 0 (early), 200 (mid), 100 (late) | 0 (baseline), 5 (early), 8, 11 (mid), 14, 17 (late) | 1088 pfu |
| Survivor 1 | 0 (early), 0 (mid), 800 (late) | 0 (early), 150 (mid), 800 (late) | 0 (baseline), 3, 6 (early), 10 (mid), 14 (late), 21 | 838 pfu |
| Survivor 2 | 0 (early), 6400 (mid), 25600 (late) | 100 (early), 12800 (mid), 3200 (late) | 0 (baseline), 4 (early), 8, 11 (mid), 14 (late), 21 | 763 pfu |
| Survivor 3 | 0 (early), 4800 (mid), 12800 (late) | 100 (early), 25600 (mid), 12800 (late) | 0 (baseline), 4 (early), 8, 11 (mid), 15 (late), 21 | 750 pfu |
| Survivor 4 | 0 (early), 3200 (mid), 12800 (late) | 100 (early), 1600 (mid), 800 (late) | 0 (baseline), 4 (early), 8, 11 (mid), 15 (late), 21 | 838 pfu |
| Survivor 5 | 0 (early), 800 (mid), 6400 (late) | 100 (early), 1600 (mid), 1600 (late) | 0 (baseline), 4 (early), 7, 11 (mid), 15 (late), 21 | 863 pfu |
| Survivor 6 | 0 (early), 200 (mid), 3200 (late) | 0 (early), 800 (mid), 800 (late) | 0 (baseline), 4 (early), 8, 11 (mid), 15 (late), 21 | 825 pfu |

**Supplementary Table 1. Individual subject BDBV GP-specific antibody titers, actual challenge doses, and sampling time points.** Disease stage is defined in parentheses. Abbreviations: PFU, plaque-forming units; AVG, average; IgG, immunoglobulin G; IgM, immunoglobulin M.
