# Supplementary material for: Bundibugyo ebolavirus Survival Is Associated with Early Activation of Adaptive Immunity and Reduced Myeloid-Derived Suppressor Cell Signaling
Source: mBio. 2021 Aug 10;12(4):e01517-21. doi: 10.1128/mBio.01517-21 (PMC8406165; doi:10.1128/mBio.01517-21)
Supplement: FIG S1 [file mbio.01517-21-sf001.docx]

**Supplementary Figure 1. Volcano plot depicting overall RNA expression changes in survivor versus fatal samples irrespective of sampling time point.** Displayed are the mean −log_10_ (*P* values) and log_2_ fold changes for each mRNA target from *n* = 10 biological replicates at each time point sampled (65 samples total). Horizontal lines within each plot indicate adjusted *P* value thresholds. Targets highlighted in blue indicate false discovery rate (FDR)-adjusted *P* values <0.10. A Benjamini–Hochberg test was employed to derive FDR-adjusted *P* values.
